# Is convenience really king? Comparative evaluation of catastrophic costs due to tuberculosis in the public and private healthcare sectors of Viet Nam: a longitudinal patient cost study

**DOI:** 10.1186/s40249-024-01196-2

**Published:** 2024-03-25

**Authors:** Hoa Binh Nguyen, Luan Nguyen Quang Vo, Rachel Jeanette Forse, Anja Maria Christine Wiemers, Huy Ba Huynh, Thuy Thi Thu Dong, Yen Thi Hoang Phan, Jacob Creswell, Thi Minh Ha Dang, Lan Huu Nguyen, Jad Shedrawy, Knut Lönnroth, Tuan Dinh Nguyen, Luong Van Dinh, Kristi Sidney Annerstedt, Andrew James Codlin

**Affiliations:** 1 National Lung Hospital, Ha Noi, Viet Nam; 2 Friends for International TB Relief, Ha Noi, Viet Nam; 3 Department of Global Public Health, WHO Collaboration Centre On Tuberculosis and Social Medicine, Karolinska Institute, Stockholm, Sweden; 4 Stop TB Partnership, Geneva, Switzerland; 5 Pham Ngoc Thach Hospital, Ho Chi Minh City, Viet Nam

**Keywords:** Tuberculosis, Patient cost, Catastrophic cost, Private sector, Comparative analysis, Viet Nam

## Abstract

**Background:**

In Viet Nam, tuberculosis (TB) represents a devastating life-event with an exorbitant price tag, partly due to lost income from daily directly observed therapy in public sector care. Thus, persons with TB may seek care in the private sector for its flexibility, convenience, and privacy. Our study aimed to measure income changes, costs and catastrophic cost incurrence among TB-affected households in the public and private sector.

**Methods:**

Between October 2020 and March 2022, we conducted 110 longitudinal patient cost interviews, among 50 patients privately treated for TB and 60 TB patients treated by the National TB Program (NTP) in Ha Noi, Hai Phong and Ho Chi Minh City, Viet Nam. Using a local adaptation of the WHO TB patient cost survey tool, participants were interviewed during the intensive phase, continuation phase and post-treatment. We compared income levels, direct and indirect treatment costs, catastrophic costs using Wilcoxon rank-sum and chi-squared tests and associated risk factors between the two cohorts using multivariate regression.

**Results:**

The pre-treatment median monthly household income was significantly higher in the private sector versus NTP cohort (USD 868 vs USD 578; *P* = 0.010). However, private sector treatment was also significantly costlier (USD 2075 vs USD 1313; *P* = 0.005), driven by direct medical costs which were 4.6 times higher than costs reported by NTP participants (USD 754 vs USD 164; *P* < 0.001). This resulted in no significant difference in catastrophic costs between the two cohorts (Private: 55% vs NTP: 52%; *P* = 0.675). Factors associated with catastrophic cost included being a single-person household [adjusted odds ratio (a*OR* = 13.71; 95% confidence interval (*CI)*: 1.36–138.14; *P* = 0.026], unemployment during treatment (a*OR* = 10.86; 95% *CI*: 2.64–44.60; *P* < 0.001) and experiencing TB-related stigma (a*OR* = 37.90; 95% *CI*: 1.72–831.73; *P* = 0.021).

**Conclusions:**

Persons with TB in Viet Nam face similarly high risk of catastrophic costs whether treated in the public or private sector. Patient costs could be reduced through expanded insurance reimbursement to minimize direct medical costs in the private sector, use of remote monitoring and multi-week/month dosing strategies to avert economic costs in the public sector and greater access to social protection mechanism in general.

**Supplementary Information:**

The online version contains supplementary material available at 10.1186/s40249-024-01196-2.

## Background

Tuberculosis (TB) is a pandemic that has ravaged public health for thousands of years [1], causing an estimated billion deaths in the past two centuries [2]. In 2022, TB was the world’s second deadliest infectious disease with 10.6 million individuals falling sick with and 1.3 million people dying of TB [3]. The disease’s devastating effects are exacerbated by its surreptitious bias towards impoverished families and individuals, granting the disease the moniker of parent, child and provider of poverty [4].

TB-affected families often face depletion of resources and savings due to the long, arduous treatment, resulting in a substantial impairment of the productivity of the patient and economic wellbeing of the TB-affected family [5–7]. Consequently, a ‘catastrophic costs’ indicator was conceived to measure the deleterious socioeconomic impact, defined as a loss of ≥ 20% of annual household income, due to an episode of TB [8]. To measure this indicator, a standardized survey instrument and guidance for data collection were developed [9]. This growing appreciation of the need to address catastrophic costs [10, 11] culminated in the inclusion of the goal of zero TB-affected households suffering from catastrophic costs by 2025 in the World Health Organization’s (WHO) End TB Strategy [12]. However, the recent estimate that 48% of individuals with TB experience catastrophic costs highlights a gaping dissonance between ambition and reality [13].

In Viet Nam, an estimated 172,000 individuals fell ill with TB, including 9200 people with drug-resistant TB in 2022, causing an estimated 13,600 deaths [14]. Quality-assured TB care is provided by the National TB Program (NTP) at District TB Units. While persons with TB receive diagnosis and treatment largely free of charge, pre-treatment services such as chest x-ray and liver function tests have to be paid out of pocket. A nationally representative patient cost survey of persons with TB receiving treatment from the NTP found that 63% of households affected by drug-susceptible TB (DS-TB) experienced catastrophic costs. The catastrophic cost incurrence was primarily driven by lost income associated with the inability to retain employment or paid leave, or having to switch to less laborious occupations with lower levels of remuneration. Root causes of these productivity losses were the TB-related disabilities and directly observed therapy (DOT) requirements tied to the quality-assured TB care provided by the NTP [15].

In response, 31% of persons with TB in Viet Nam seek care in the private sector. Despite concerns about the quality or safety of care, and potential supply-induced demand [16–18], the greater privacy to protect from stigma and related negative social consequences especially for women [19–22] along with the convenience of self-administered treatment, multi-day dosing, shorter wait times and after-hour consultations are perceived to outweigh high out-of-pocket treatment costs [23–26]. However, to date there have been no studies to verify this perceived trade-off by directly comparing catastrophic cost incurrence in TB-affected households between people receiving care from the private sector versus the NTP. Our study aimed to measure income changes, costs and catastrophic cost incurrence among TB-affected households in the public and private sector.

## Methods

### Study design

This was a prospective cohort study to measure the comparative patient costs and rates of catastrophic cost incurrence among affected families due to an episode of DS-TB in public and private sectors. Data were collected using a localized, longitudinal adaptation of the WHO patient cost survey tool.

### Study setting

The study was conducted in Ha Noi, Hai Phong and Ho Chi Minh City (HCMC). These high TB burden, urban provinces had a combined population of 19.1 million persons per the latest census and according to NTP surveillance notified 23,502 persons with DS-TB in 2019. Since 2017, the Viet Nam NTP has implemented a private sector engagement model with an implementation partner, Friends for International TB Relief, serving the role of an intermediary agency to collect, verify and notify private TB treatment data [16]. This collaboration enabled the patient cost comparisons between private and public cohorts.

### Study population and eligibility

The study population consisted of persons with DS-TB taking and completing treatment with the NTP or a private healthcare provider. Recruitment occurred between October 2020 and March 2022 and employed a continuous sampling strategy for the private cohort. Participants for the public cohort were recruited to match with private participants in terms of residing district and treatment initiation date. All treatment-naïve persons aged 18 years or older with pulmonary DS-TB, residence in the study provinces and providing informed consent to participate were included. Persons already participating in or with a household member participating in another patient cost survey were excluded to avoid double counting this household in the overall dataset, and avoid bias from overstating the risk of catastrophic costs.

### Data sources and collection

Our study employed the WHO patient cost survey tool, adapted for longitudinal data collection and for local conditions as detailed elsewhere [27]. Briefly, the tool assessed participant characteristics, financial and economic costs, and socioeconomic impact due to the episode of TB. The latter included changes in employment status, food insecurity, productivity loss, social exclusion and use of coping strategies [9]. In terms of localization, the tool was translated and the survey’s asset list was expanded with items relevant for the urban Vietnamese context. For the longitudinal adaptation, questions concerning pre-treatment costs, social health insurance (SHI) status and asset ownership were omitted during the second and third interviews. To reduce recall bias, participants were surveyed at three separate time points. In accordance with national TB treatment guidelines, uncomplicated DS-TB treatment consists of a two-month intensive phase, followed by a four-month continuation phase. The first survey took place within the intensive phase of treatment, but after two weeks from initiation. The second survey was conducted within two months after the participant entered the continuation phase of treatment. The last survey took place within two months of treatment completion.

Recruitment in the public sector was done by study staff situated at the public TB care facilities upon treatment initiation. Private sector recruitment occurred based on introduction and referral by the private provider with their client’s consent. Interviews were typically conducted face-to-face at the Lung Hospital, District TB Unit, commune health station, patient’s house or other places convenient for the participant. From 2021, interviews were conducted primarily via phone to sustain recruitment and ensure follow-up during periods of social distancing in response to the COVID-19 pandemic [28]. Paper-based consent forms were collected from all participants regardless of the interview modality. Participants were asked to produce receipts from all healthcare interactions at their second and third interview to reduce recall and social desirability bias. Data were collected on paper and audio recorded. Paper surveys were digitized using the Organizational Network Analysis data survey tool (Ona, Nairobi, Kenya). On a monthly basis, five percent of digital surveys were randomly selected and verified by another interviewer using the audio recordings.

### Statistical analyses

Demographic, socioeconomic, clinical and behavioral characteristics as well as health seeking behaviors were described by public and private cohort. Categorical variables were tabulated by frequencies and proportions; continuous variables by mean and standard deviation, or median and interquartile range based on their distribution. To analyze differences, Chi-squared, Fisher's exact, Student’s *t* or Wilcoxon rank-sum tests were performed as applicable.

Household incomes pre-treatment, during the intensive phase, the continuation phase and at the end of treatment for both cohorts were tabulated by mean and standard deviation, as well as median and interquartile range, and compared by Wilcoxon rank-sum test due to their right skewed distribution.

Patient cost calculations followed WHO guidelines [9]. Direct medical costs encompassed consultations, diagnostic tests, hospitalizations and medications. Direct non-medical expenses encompassed expenditure related to food, travel and accommodation arising from medical visits, supplements and loan interest. Indirect costs were defined as reported income loss during treatment only and were calculated using the output approach with caregiver time loss excluded to match the national patient cost survey [15]. We calculated mean and median direct medical, direct non-medical and indirect costs for the entire episode of TB, as well as disaggregated into pre-treatment and treatment phases. Given large standard deviations, we used the Wilcoxon rank-sum test to analyze differences in costs between the two cohorts. We disaggregated total treatment costs by main cost components (medical, non-medical and indirect) and direct medical costs by unplanned health care visits and hospitalizations, drug pick-ups, and follow-up appointments. These cost breakdowns were compared across cohort using *t*-tests for proportions.

Catastrophic cost was defined as total costs exceeding 20% of the annual household income prior to diagnosis [9]. The catastrophic cost estimates for the two cohorts were compared using *χ*^2^ tests. We fitted univariate and multivariate logistic regression models to assess the association between catastrophic cost incurrence as the primary outcome and survey cohort as the primary exposure. Secondary covariates were included to adjust for confounding included demographic, socioeconomic, behavioral and health-seeking factors based on prior research [29–31]. A post-hoc analysis to investigate the association of household income and patient costs in the private sector did not yield relevant associations (Additional file 1).

All costs were collected in Viet Nam Dong (VND) and converted to US dollars (USD) using the average 2020–2022 exchange rates reported by XE.com for the study period (VND 1 = USD 0.000043). Hypotheses were two-tailed and *P*-values below 0.05 were considered statistically significant. Data were analyzed using Stata v17 (Stata Corp, College Station, TX, USA).

## Results

### Participant characteristics and health-seeking

We identified 294 persons TB treated in the private sector and 90 by the NTP. Of these, we recruited 64 participants in each of the private (64/294 = 22%) and NTP (64/90 = 71%) survey cohorts. There were 14 (14/64 = 22%) and 4 (4/64 = 6%) participants in the respective cohorts who did not complete all of the survey milestones. Therefore, the final study sample consisted of 50 private sector and 60 NTP participants (Table 1).

**Table 1:** Demographic, socioeconomic, clinical and behaviorial characteristics of the participant sample bifurcated by cohort receiving treatment in the public and private sector

	All (*n* = 110)	Private (*n* = 50)	NTP (*n* = 60)	*P*-value^¥^
Demographic characteristics				
Male, *n* (%)	79 (72)	33 (66)	46 (77)	0.216
Age, mean (*SD*), years	44.4 (15.9)	44.9 (17.5)	44.1 (14.6)	0.777
Socio-economic factors				
Single-person household*, n* (%)	8 (7)	2 (4)	6 (10)	0.228
Household’s primary source of income pre-TB, *n* (%)	48 (44)	18 (36)	30 (50)	0.140
Complete secondary education, *n* (%)	56 (51)	31 (62)	25 (42)	0.034
Employment pre-TB, *n* (%)				0.782
Unemployed	14 (13)	7 (14)	7 (12)	–
Formally employed	28 (25)	9 (18)	18 (30)	–
Informally employed	36 (33)	17 (34)	19 (32)	–
Retired	10 (9)	6 (12)	4 (7)	–
Student	4 (4)	2 (4)	2 (3)	–
Housework	3 (3)	2 (4)	1 (2)	–
Other	16 (15)	7 (14)	9 (15)	–
Unemployed during TB treatment, *n* (%)	45 (41)	17 (34)	28 (47)	0.178
Health insurance, *n* (%)	94 (85)	42 (84)	52 (87)	0.693
Experienced stigma, *n* (%)	14 (13)	4 (8)	10 (17)	0.174
Clinical factors				
Previous TB episodes, *n* (%)	7 (6)	3 (6)	4 (6)	0.887
Bacteriologically confirmed, *n* (%)	77 (70)	40 (80)	37 (62)	0.037
Presence of comorbidity, *n* (%)	85 (77)	42 (84)	43 (72)	0.124
Behavioral risk factors				
Daily alcohol consumption, *n* (%)	6 (5)	4 (8)	2 (3)	0.283
Daily smoking, *n* (%)	19 (17)	6 (12)	13 (22)	0.182
Health-seeking behaviors				
Treatment delay (> 4 weeks from symptom onset to treatment initiation), *n* (%)	82 (75)	41 (82)	41 (68)	0.101
Time between onset of TB symptoms and treatment initiation, median (IQR), weeks	8 (4–16)	8 (5–15)	6 (4–17)	0.552
Number of visits pre-treatment, median (IQR)	5 (3–9)	5 (3–7)	6 (4–9)	0.046
Hospitalization during treatment, *n* (%)	27 (25)	8 (16)	19 (32)	0.057

^¥^Chi-square, Fischer Exact (any cell with *n* < 5 in the contingency table) for proportions, Wilcoxon rank-sum for medians,* t*-test for means; *TB* Tuberculosis; *SD* Standard deviation*; IQR* Interquartile range; *NTP* National TB Program

Most participants were male (79/110 = 72%), with a mean age of 44.4 years. About 51% reported completion of secondary schooling, 44% indicated that they were the main earners in their household and 85% reported SHI coverage. While 13% were unemployed before treatment initiation, this proportion increased to 41% during treatment. Only 6% reported a history of TB, but the majority (77%) reported a comorbidity. About 5% of participants reported daily alcohol consumption, while 17% were daily smokers. In terms of health-seeking, the median diagnostic delay was eight weeks between the onset of symptoms and treatment initiation.

While most of the sample was homogenous across the two cohorts, significantly more participants from the private sector cohort completed secondary education compared to the NTP cohort (62% vs 42%; *P* = 0.034). There were also significantly more participants with bacteriologic confirmation in the private sector cohort (80% vs 62%; *P* = 0.037). Similarly, participants from the private sector cohort visited more healthcare providers prior to treatment (5 vs 6, *P* = 0.046).

### Household income

The pre-treatment median monthly household income was USD 678 [Inter-quartile range (IQR): USD 430–1032] across all participants (Table 2). Household incomes fell by over 30% to USD 471 (IQR: USD 322–903) and USD 473 (IQR: USD 292–882) during the intensive and continuation phases, respectively, and rose to USD 602 (IQR: USD 280–860) by the end of treatment, representing 89% of the pre-TB earnings. The median household income was significantly higher in the private sector cohort prior to treatment (USD 868 vs USD 578; *P* = 0.010) and during the intensive phase of treatment (USD 763 vs USD 419; *P* = 0.003), but income differences in the continuation phase were not significant. By the end of treatment significant differences in income between the private sector and NTP cohorts were restored (USD 710 vs USD 464; *P* = 0.006).

**Table 2:** Median monthly household income before, during and by the end of treatment

	All (*n* = 110)		Private (*n* = 50)		NTP (*n* = 60)		*P*-value^¥^
	Mean (*SD*)	Median (IQR)	Mean (*SD*)	Median (IQR)	Mean (*SD*)	Median (IQR)	
Pre-treatment	1006 (1250)	678 (430–1032)	1308 (1652)	868 (495–1247)	753 (692)	578 (430–849)	0.010
Intensive phase	860 (1228)	471 (322–903)	1166 (1591)	763 (421–1161)	606 (734)	419 (258–692)	0.003
Continuation phase	776 (932)	473 (292–882)	969 (1178)	602 (387–1008)	615 (630)	441 (252–774)	0.070
End of treatment	764 (887)	602 (280–860)	975 (1097)	710 (366–1032)	587 (621)	464 (237–710)	0.006

^¥^Wilcoxon rank-sum test on median values; *NTP* National TB Program; *SD* Standard deviation; *IQR* Interquartile range

### Costs of TB care

**Fig. 1:**
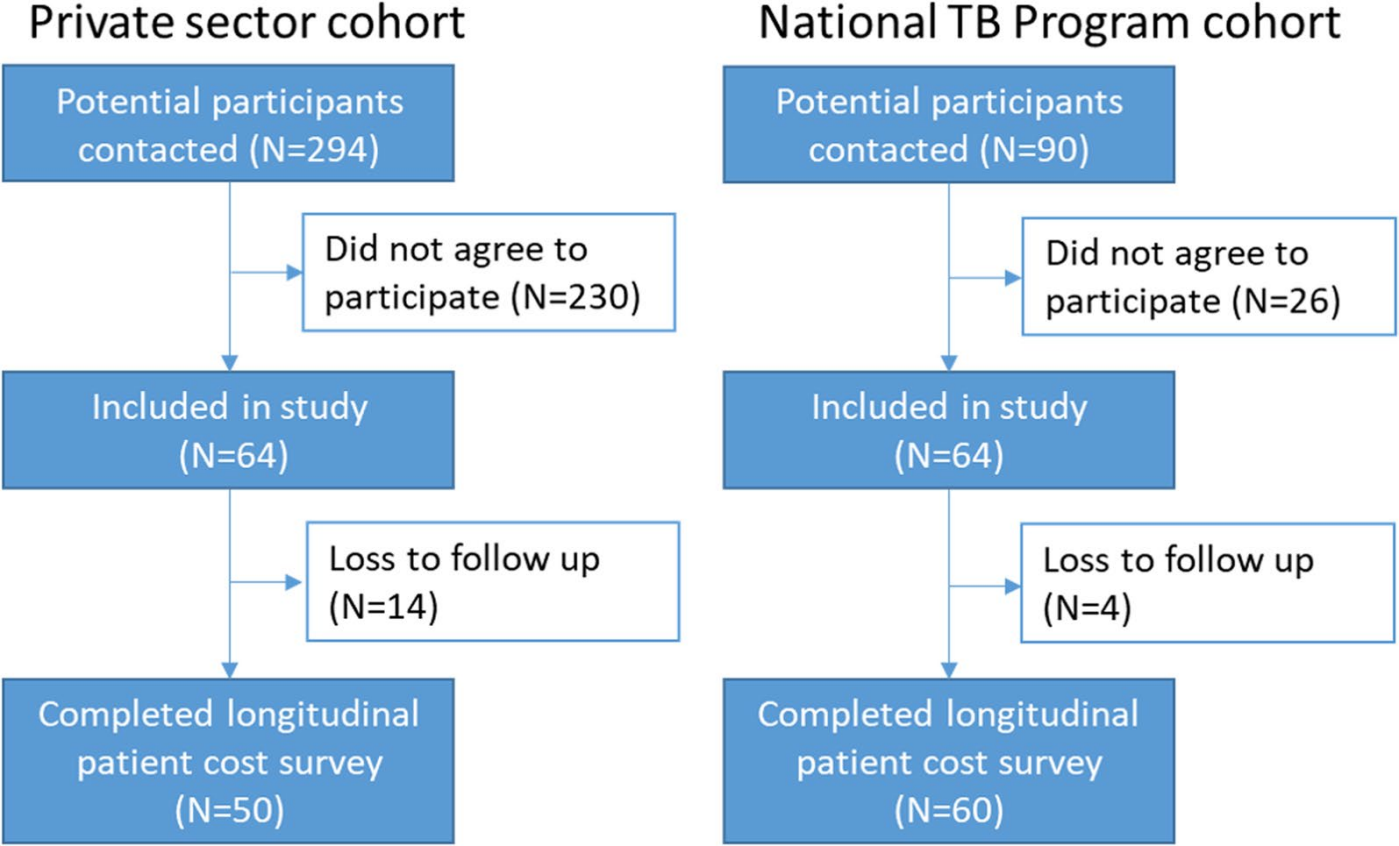
Recruitment flow chart of the study by cohort

The median total costs of an episode of DS-TB across the sample were USD 1726 (IQR: USD 879–2820) (Table 3). Of these, median pre-treatment costs were USD 151 (IQR: USD 72–236) and consisted mainly of direct medical costs of USD 138 (IQR: USD 55–212). Median treatment costs were USD 1541 (IQR: USD 737–2477), driven primarily by indirect costs with a median of USD 585 (IQR: USD 0–1548) (Fig. 1).

**Table 3:** Direct and indirect patient costs before and during treatment

	All (*n* = 110)		Private (*n* = 50)		NTP (*n* = 60)		*P*-value^¥^
	Mean (*SD*)	Median (IQR)	Mean (*SD*)	Median (IQR)	Mean (*SD*)	Median (IQR)	
Pre- & treatment costs							
Direct medical costs	599 (630)	402 (147–839)	974 (696)	754 (508–1223)	287 (334)	164 (61–398)	< 0.001
Direct non-medical costs	456 (493)	355 (196–542)	539 (670)	398 (133–648)	388 (255)	345 (233–480)	0.737
Indirect costs	1347 (2938)	585 (0–1548)	1732 (4121)	710 (0–1806)	1025 (1270)	578 (0–1527)	0.954
Total pre- & treatment costs	2402 (3257)	1726 (879–2820)	3246 (4481)	2075 (1229–3481)	1700 (1349)	1313 (652–2174)	0.005
Pre-treatment costs							
Direct medical costs	221 (343)	138 (55–212)	268 (441)	153 (85–213)	181 (229)	99 (43–202)	0.028
Direct non-medical costs	28 (55)	9 (4–25)	21 (31)	6 (3–22)	34 (68)	9 (5–30)	0.053
Total pre-treatment costs	248 (371)	151 (72–236)	288 (455)	166 (92–235)	248 (371)	114 (53–245)	0.065
Treatment costs							
Direct medical costs	379 (477)	198 (15–621)	707 (476)	609 (350–970)	106 (253)	16 (1–98)	< 0.001
Direct non-medical costs	429 (486)	329 (153–530)	519 (659)	384 (131–637)	354 (251)	315 (188–429)	0.560
Indirect costs	1347 (2938)	585 (0–1548)	1732 (4121)	710 (0–1806)	1025 (1270)	578 (0–1527)	0.954
Total treatment costs	2154 (3180)	1541 (737–2477)	2957 (4390)	1864 (969–2967)	1485 (1293)	1111 (527–1995)	0.004

^¥^Wilcoxon rank-sum test on median values; *NTP* National TB Program; *SD* Standard deviation; *IQR* Interquartile range

**Fig. 2:**
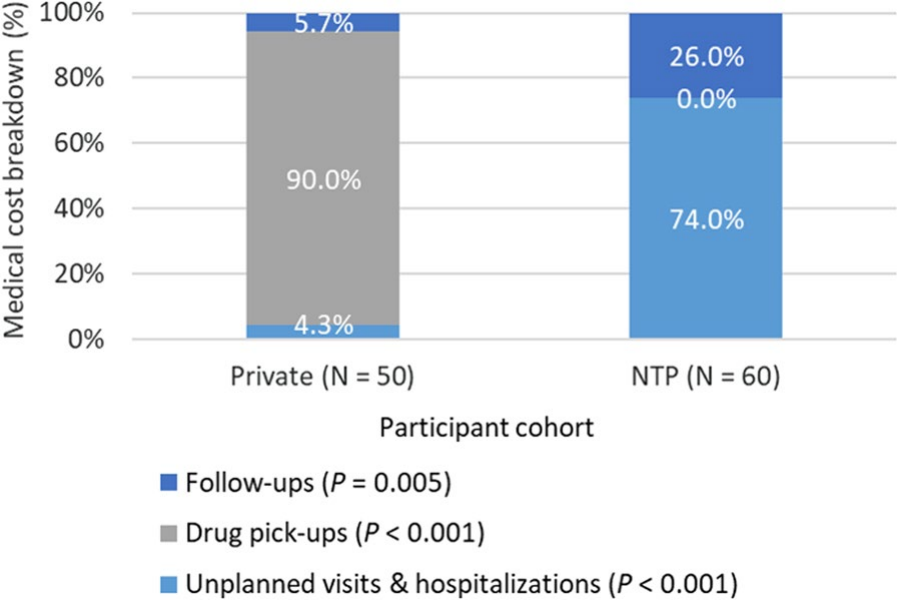
Treatment medical cost breakdown, private (USD 609) vs. National TB Program cohorts (USD 16). *NTP* National TB Program; *P*-values calculated using 2-sample *t*-test of proportions

**Fig. 3:**
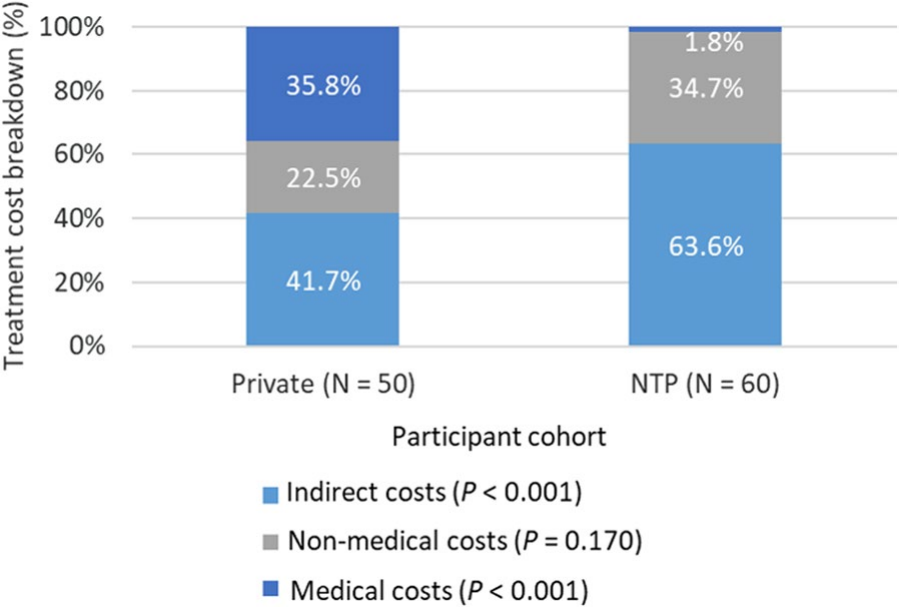
Treatment total cost breakdown, private (USD 1864) vs. National TB Program cohorts (USD 1111). *NTP* National TB Program; *P*-values calculated using 2-sample* t*-test of proportions

When comparing private sector and NTP cohorts, median total costs in the private sector cohort were significantly higher (USD 2075 vs USD 1313; *P* = 0.005). This was primarily driven by the significant differences in direct medical costs (USD 754 vs USD 164; *P* < 0.0001) in both the pre-treatment (USD 153 vs USD 99; *P* = 0.028) and treatment phases (USD 609 vs USD 16; *P* < 0.001). Specifically, the main cost driver was the cost of drugs, which comprised 90% direct medical costs within the private sector cohort (Fig. 2). Between the private sector and NTP cohorts, there were no significant differences in median direct non-medical costs (USD 398 vs USD 345; *P* = 0.737) and indirect costs from lost salaries and wages (USD 710 vs USD 578; *P* = 0.954). However, indirect costs comprised a significantly higher portion of total costs for the NTP cohort (64% vs 42%; *P* = 0.021) due to the larger share of direct medical costs in the private sector cohort (Fig. 3).

### Catastrophic costs and associated risks

Catastrophic cost incurrence in the private sector and NTP cohorts were 52% and 55%, respectively (Table 4). There was no evidence of a statistical difference in the risk of catastrophic cost incurrence in either cohort [adjusted Odds Ratio (a*OR*) = 1.26; 95% *CI*: 0.42–3.77; *P* = 0.675]. Instead, risk factors associated with catastrophic cost incurrence include being a single-person household (a*OR* = 13.71; 95% *CI*: 1.36–138.14; *P* = 0.026), unemployment during treatment (a*OR* = 10.86; 95% *CI*: 2.64–44.60; *P* < 0.001) and experiencing TB-related stigma (a*OR* = 37.90; 95% *CI*: 1.72–831.73; *P* = 0.021).

**Table 4:** Catastrophic cost incurrence and associations with participant characteristics

	Catastrophic cost incurrence *n*_*C*_*/n*_*T*_^†^ (%^‡^)	Crude *OR*^¶^ [95% *CI*]	*P*-value^¥^	Adjusted *OR*^§^ [95% *CI*]	*P*-value^¥^
Cohort					
NTP	33/60 (55)	Ref.		Ref.	
Private	26/50 (52)	0.89 [0.42–1.88]	0.753	1.26 [0.42–3.77]	0.675
Demographic factors					
Gender					
Male	43/79 (54)	Ref.	–	Ref.	–
Female	16/31 (52)	0.89 [0.39–2.05]	0.790	0.78 [0.20–3.01]	0.723
Age, years					
Median age (IQR)	47 (40–59)	1.03 [1.00–105]	0.031	0.99 [0.94–1.03]	0.548
Socioeconomic factors					
Single-person household					
No	53/102 (52)	Ref.	–	Ref.	–
Yes	6/8 (75)	2.77 [0.53–14.40]	0.225	13.71 [1.36–138.14]	0.026
Completed secondary education					
No	33/54 (61)	Ref.	–	Ref.	–
Yes	26/56 (46)	0.55 [0.26–1.18]	0.124	1.88 [0.52–6.86]	0.337
Employment pre-TB					
Unemployed	10/14 (71)	Ref.		Ref.	
Formal paid work	8/27 (30)	0.17 [0.04–0.70]	0.014	0.34 [0.04–2.64]	0.303
Informal paid work	22/36 (61)	0.63 [0.16–2.40]	0.497	2.98 [0.45–19.66]	0.257
Retired	6/10 (60)	0.60 [0.11–3.34]	0.560	4.85 [0.33–70.55]	0.248
Student	0/4 (0)	1.00	N.A	1.00	N.A
Housework	1/3 (33)	0.20 [0.01–2.88]	0.237	0.70 [0.02–21.35]	0.248
Other	12/16 (75)	1.20 [0.24–6.06]	0.825	6.9 [0.60–80.30]	0.121
Unemployed during treatment					
No	24/65 (37)	Ref.	–	Ref.	–
Yes	35/45 (78)	5.98 [2.52–14.20]	< 0.001	10.86 [2.64–44.61]	< 0.001
Household’s primary source of income pre-TB					
No	32/62 (52)	Ref.	–	Ref.	–
Yes	27/48 (56)	1.21 [0.57–2.57]	0.629	1.47 [0.46–4.66]	0.515
Health insurance					
No	7/16 (44)	Ref.	–	Ref.	–
Yes	52/94 (55)	1.49 [0.55–4.63]	0.394	4.15 [0.63–27.38]	0.139
Experienced stigma					
No	47/96 (50)	Ref.	–	Ref.	–
Yes	12/14 (86)	6.26 [1.33–29.46]	0.02	37.9 [1.72–831.73]	0.021
Clinical factors					
Previous TB episodes					
No	55/103 (53)	Ref.	–	Ref.	–
Yes	4/7 (57)	1.16 [0.25–5.46]	0.848	1.94 [0.22–16.92]	0.548
Bacteriologically confirmed					
No	17/33 (52)	Ref.	–	Ref.	–
Yes	42/77 (55)	1.13 [0.50–2.56]	0.770	1.61 [0.49–5.32]	0.433
Presence of co-morbidity					
No	17/25 (68)	Ref.	–	Ref.	–
Yes	42/85 (49)	0.46 [0.18–1.18]	0.106	0.43 [0.11–1.63]	0.216
Behavioral factors					
Daily alcohol consumption					
No	54/104 (52)	Ref.	–	Ref.	–
Yes	5/6 (83)	4.63 [0.52–41.01]	0.170	3.41 [0.24–47.56]	0.361
Daily smoking					
No	49/91 (54)	Ref.	–	Ref.	–
Yes	10/19 (53)	0.95 [0.35–2.56]	0.923	0.25 [0.04–1.46]	0.124
Health-seeking factors					
Treatment delay					
No	15/28 (54)	Ref.	–	Ref.	–
Yes	44/82 (54)	1.00 [0.42–2.37]	0.994	1.22 [0.32–4.63]	0.767
Hospitalization					
No	42/83 (51)	Ref.	–	Ref.	–
Yes	17/27 (63)	1.66 [0.68–4.05]	0.266	2.64 [0.60–8.50]	0.226

^†^Ratio of the number of participants experiencing catastrophic costs (*n*_*C*_) divided by the number of participants belonging to the category in that line (*n*_*T*_). For example, there were 43 participants experiencing catastrophic costs (*n*_*C*_) among 79 total male participants (*n*_*T*_); ^‡^Catastrophic cost threshold at 20% based on the output approach; ^¶^Univariate logistic regression; ^§^Multivariate logistic regression incorporating all shown participant covariates; ^¥^Wald test. *TB* Tuberculosis; *NTP* National TB Program

## Discussion

Our study found no difference in catastrophic cost incurrence in public and private sector TB treatment. Surprisingly, we also did not detect a difference in indirect costs, which disproves our hypothesis that the lack of DOT in private sector care can alleviate costs from lost salaries and wages. Conversely, as expected we measured high direct medical costs before and during treatment in the private sector, largely arising from drug pick-ups, as well as higher pre-treatment household incomes among private sector participants to help absorb the higher treatment costs. Overall, our study highlighted that an episode of TB in Viet Nam represents a costly life-event, irrespective of whether treatment is sought in public or private sectors, with catastrophic costs mainly driven by economic and social factors.

The rates of catastrophic cost incurrence measured on our study (52–55%) were concordant with rates measured among DS-TB patients receiving NTP care on prior studies conducted in Viet Nam at national and sub-national levels (30–63%). This concordance also applies to previously reported median total costs of an episode of DS-TB (USD 894–1779), median indirect costs (USD 460–925) and the percentage of indirect costs as a proportion of total (48–68%), strengthening the generalizability of our results in the Vietnamese context [27, 32, 33].

Our regression analysis showed that catastrophic cost incurrence was driven mainly by socioeconomic risk factors, such as being a single income household and becoming unemployed during TB treatment. This association between income insecurity and susceptibility to catastrophic costs is well documented in Viet Nam and other high burden settings [31, 34, 35]. As such, expansion of access, coverage and level of support of social protection mechanisms in TB-endemic countries remains urgently needed [36]. One such example is the charity fund for TB patients, called Patient Support to Fight TB (PASTB), established by the Viet Nam NTP in response to the national patient cost survey results. PASTB has financed the purchase of SHI coverage and unreimbursed expenses related to TB care such as chest x-rays or liver function tests [37]. Beyond these short-term mitigation efforts, the NTP has sought collaborations with the Ministry of Health and Ministry of Labor, Invalids and Social Affairs to explore adaptation measures, such as making existing social protection schemes accessible to persons with TB, linking TB survivors with vocational training and exploring avenues for better job protection and worker’s rights [38]. In the short-term, alternative indirect measures could include scaling up active case finding and community-based interventions to alleviate the economic and social burden on TB-affected families and individuals [39, 40].

Another risk factor of catastrophic cost incurrence was patient-experienced stigma. Studies have reported that public sector care and the associated DOT requirement may intensify stigma [41, 42]. Consequently, there is a growing momentum towards people-centered care that addresses social determinants, including stigmatization [43]. As part of this movement, demands have included the phase out of traditional DOT, which has constituted a pillar of public sector patient management over the last three decades [44–46]. Removing the DOT requirement could both reduce stigma and alleviate much of the indirect cost burden in the public sector, which represented 52% of total treatment costs within the NTP cohort and was equal to the average monthly household income before the episode of TB.

While the lack of DOT has long represented a core value proposition of the private sector to clients [19, 47], it also constitutes one of its core criticisms from a public health perspective, due to suspicions of fueling drug resistance [17, 48]. Thus, it may be helpful to offer an alternative to ensure that the quality of care is maintained. Many process alternatives to facility-based DOT have been deemed effective for patient care [46, 49]. Specifically, these alternatives include community-based DOT [50–52], home delivery and multi-period dispensing for self-administered therapy [53, 54]. More recently, digital adherence technologies (DAT) have emerged [55–57]. These include video-DOT [58, 59], SMS-based remote monitoring tools, such as 99DOTS [60], and Medication Event Reminder Monitors [61]. These tools remain underutilized given the limited, discordant evidence on the impact of DATs on clinical outcomes, incremental treatment costs and patient-centeredness of care [62–65].

With respect to optimizing private sector TB care, direct medical costs could be shifted to public health financing schemes. Viet Nam has set ambitious targets of achieving Universal Health Coverage through its national SHI scheme [66]. In doing so, Viet Nam has embarked on transitioning major public health programs for HIV and TB to SHI financing [67, 68]. However, the TB transition is in its infancy and thus continues to face challenges [69] and the current national SHI scheme offers suboptimal protection [70]. Nevertheless, the principle could conceivably be applied to the private sector as well to reduce the financial burden of treatment for patients [71, 72]. This is particularly needed for informally employed individuals who comprised one-third of our sample. Their options for treatment tend to be restricted by their limited job security, which may force them to seek out costly private sector care as a form of social protection, despite the premium it commands. It is noteworthy that regional precedence exists. In the healthcare systems of some Asian countries, privatization of TB care and pay-for-performance schemes have task shifted a portion of the TB caseload to the private sector [73, 74].

A key limitation of our study was the small sample size of persons with TB receiving private sector care who agreed to participate. This resulted in a potential underpowering of statistical comparisons leading to missed distinctions between the two cohorts. Furthermore, a core value proposition of private TB treatment is the privacy offered to patients unwilling to be exposed to the stigma attached to the disease, particularly among women, so that our results contain an inherent selection bias [75, 76]. Lastly, this study was set in three municipal provinces with a high degree of urbanization, thus limiting generalizability to rural settings, where travel times and transport costs would likely be very different. The timing of the data collection, much of which occurred at the height of the COVID-19 pandemic, may have further introduced bias, as external factors other than TB may have affected drivers of catastrophic costs.

This was the first direct comparison of catastrophic cost incurrence in private versus public sector patients. More evidence is undoubtedly needed to elucidate the underlying differences between private versus NTP care, including job security and social protection, the consequent complex choices persons with TB have to make in light of the prevailing information asymmetry and threat of misinformation, and the socioeconomic consequences they have to bear. However, there is little doubt that the question of how to achieve zero TB-affected families suffering catastrophic costs must be addressed to ensure greater health equity, effective abatement of the deleterious socioeconomic consequences and to end TB both as a public health emergency and a catastrophic life-event for affected families.

## Conclusions

High costs for patients and affected families remain a major barrier to global ambitions to end TB. In their personal calculus, each person with TB needs to make the choice of treatment provider, public or private, that offers the greatest perceived benefit. Currently, TB-affected persons in Viet Nam either face high time loss and foregone income with the NTP or high out-of-pocket treatment costs with private providers. It is therefore imperative to explore ways to invert the current paradigm and enable persons with TB to have access to several affordable treatment options. This particularly applies to TB-affected households that typically are poorer than the general population. Changes in policies and practice, such as expansion of SHI reimbursement for private TB care services or introduction of remote adherence monitoring by the NTP, may alleviate the socioeconomic burden for TB-affected individuals and families, accelerate reductions in catastrophic costs towards achieving End TB Strategy goals, and produce greater equity in health.

### Supplementary Information

**Additional file 1. **Supplementary material and table.

## Data Availability

The data that support the findings of this study are available from the Viet Nam National Lung Hospital/NTP, Ha Noi Lung Hospital, Hai Phong Lung Hospital and Pham Ngoc Thach Hospital. However, restrictions apply to the availability of these data, which include programmatic clinical patient information, and so are not publicly available. Data can be made available from the authors upon reasonable request and with permission of the relevant government authorities listed above.

## Abbreviations

DAT: Digital adherence technology
DOT: Directly observed therapy
HCMC: Ho Chi Minh City
IQR: Inter-quartile range
NTP: National TB program
(a)*OR*: (Adjusted) odds ratio
*SD*: Standard deviation
SHI: Social health insurance
(DS-)TB: (Drug-susceptible) tuberculosis
VNĐ: Viet Nam Dong
WHO: World Health Organization
