# Supplementary material for: Is convenience really king? Comparative evaluation of catastrophic costs due to tuberculosis in the public and private healthcare sectors of Viet Nam: a longitudinal patient cost study
Source: Infect Dis Poverty. 2024 Mar 25;13:27. doi: 10.1186/s40249-024-01196-2 (PMC10962209; doi:10.1186/s40249-024-01196-2)
Supplement: Supplementary file 1 — Additional file 1. Supplementary material and table. [file 40249_2024_1196_MOESM1_ESM.docx]

# Supplementary Information

## Supplementary results

**Sub-analysis of association between household income and patient costs in private sector**

**Methods**: We fitted univariate logistic regressions to examine the relationship between catastrophic costs (CC) incurrence and household income pre-TB quintiles, quartiles and tertiles as well as using household income as a continuous variable. Linear regressions were applied to examine the relationship between household income groups and total as well as direct costs. The results of the univariate regressions can be found in below. Fitting saturated multivariate regressions with the household income groups lead to model instability due to the limited sample size (p=50).

**Table S1. Association of household income pre-TB with catastrophic cost incurrence, total costs and direct costs in the private sector cohort**

| HH Income | CC Incurrence§ | | Total costs (USD)‡ | | Direct costs (USD)‡ | |
| --- | --- | --- | --- | --- | --- | --- |
| *pre-TB [IQR]* | *OR [95% CI]* | *p* | *Coeff. [95% CI]* | *p* | *Coeff. [95% CI]* | *p* |
| Quintiles (poorest to richest) | | | | | | |
| 1^st^ | Ref | - | Ref | - | Ref | - |
| 2^nd^ | 0.38 [0.05-2.77] | 0.337 | 316 [(-3538)-4170] | 0.869 | - 306 [(-1289)-677] | 0.534 |
| 3^rd^ | 0.11 [0.01-0.83] | **0.033** | 487 [(-3368)-4341] | 0.800 | -610 [(-1593)-373] | 0.218 |
| 4^th^ | 0.58 [0.07-4.56] | 0.608 | 1633 [(-2222)-5487] | 0.398 | -49 [(-1032)-934] | 0.921 |
| 5^th^ | 0.06 [0.01-0.56] | **0.013** | 4861 [1006-8715] | **0.015** | -33 [(-1016)-950] | 0.946 |
| Quartiles | | | | | | |
| 1^st^ | Ref | - | Ref | - | Ref | - |
| 2^nd^ | 0.21 [0.04-1.20] | 0.080 | -270 [(-3619)-3079] | 0.872 | -629 [(-1469)-211] | 0.138 |
| 3^rd^ | 0.35 [0.06-1.90] | 0.223 | 528 [(-2754)-3809] | 0.748 | -589 [(-1412)-234] | 0.156 |
| 4^th^ | 0.15 [0.03-0.87] | **0.035** | 4600 [1250-7959] | **0.008** | 187 [(-653)-1027] | 0.657 |
| Tertiles | | | | | | |
| 1^st^ | Ref | - | Ref | - | Ref | - |
| 2^nd^ | 0.37 [0.09-1.52] | 0.168 | 353 [(-25570)-3274] | 0.809 | -395 [(-1132)-342] | 0.286 |
| 3^rd^ | 0.25 [0.06-1.07] | 0.062 | 3765 [798-6732] | **0.014** | 155 [(-593)-903] | 0.679 |
| As continuous variable | | | | | | |
| Univariate | 0.99 [0.99- 1.00] | 0.174 | 1.85 [1.28-2.43] | **<0.001** | 0.03 [(-0.15)-0.22] | 0.710 |
| Multivariate¥ | 1.00 [0.99-1.00] | 0.966 | 2.12 [1.35-2.88] | **<0.001** | 0.14 [(-0.12)-0.39] | 0.281 |
| § *Univariate logistic regression;* ‡ *Univariate linear regression;* ¥  *Saturated multivariate linear regression; IQR: Interquartile range; OR: Odds ratio; CI: Confidence Interval; Coeff.: Coefficient; significant p-values marked in bold* | | | | | | |

**Results and Interpretation:** The highest income households showed lower catastrophic cost incurrence. The highest earning households also experienced higher total costs driven by income loss, i.e., economic/opportunity cost. This may explain why the difference in CC amongst household income groups was not as pronounced. There were no significant differences in regard to direct costs (i.e. through differentiated pricing by the doctors). When using HH income as a continuous variable both univariate and multivariate regressions produced similar results. Specifically, higher household incomes led to an increase in total costs, but there was no association with CC incurrence of direct costs.
